# Bioinformatic Analysis Reveals Archaeal tRNA^Tyr^ and tRNA^Trp^ Identities in Bacteria

**DOI:** 10.3390/life7010008

**Published:** 2017-02-21

**Authors:** Takahito Mukai, Noah M. Reynolds, Ana Crnković, Dieter Söll

**Affiliations:** 1 Department of Molecular Biophysics and Biochemistry, Yale University, New Haven, CT 06520, USA; takahito.mukai@yale.edu (T.M.); noah.reynolds@yale.edu (N.M.R.); ana.crnkovic@yale.edu (A.C.); 2 Department of Chemistry, Yale University, New Haven, CT 06520, USA

**Keywords:** tRNA, aaRS, genetic code, evolution, lateral gene transfer

## Abstract

The tRNA identity elements for some amino acids are distinct between the bacterial and archaeal domains. Searching in recent genomic and metagenomic sequence data, we found some candidate phyla radiation (CPR) bacteria with archaeal tRNA identity for Tyr-tRNA and Trp-tRNA synthesis. These bacteria possess genes for tyrosyl-tRNA synthetase (TyrRS) and tryptophanyl-tRNA synthetase (TrpRS) predicted to be derived from DPANN superphylum archaea, while the cognate tRNA^Tyr^ and tRNA^Trp^ genes reveal bacterial or archaeal origins. We identified a trace of domain fusion and swapping in the archaeal-type TyrRS gene of a bacterial lineage, suggesting that CPR bacteria may have used this mechanism to create diverse proteins. Archaeal-type TrpRS of bacteria and a few TrpRS species of DPANN archaea represent a new phylogenetic clade (named TrpRS-A). The TrpRS-A open reading frames (ORFs) are always associated with another ORF (named ORF1) encoding an unknown protein without global sequence identity to any known protein. However, our protein structure prediction identified a putative HIGH-motif and KMSKS-motif as well as many α-helices that are characteristic of class I aminoacyl-tRNA synthetase (aaRS) homologs. These results provide another example of the diversity of molecular components that implement the genetic code and provide a clue to the early evolution of life and the genetic code.

## 1. Introduction

**Figure 1:**
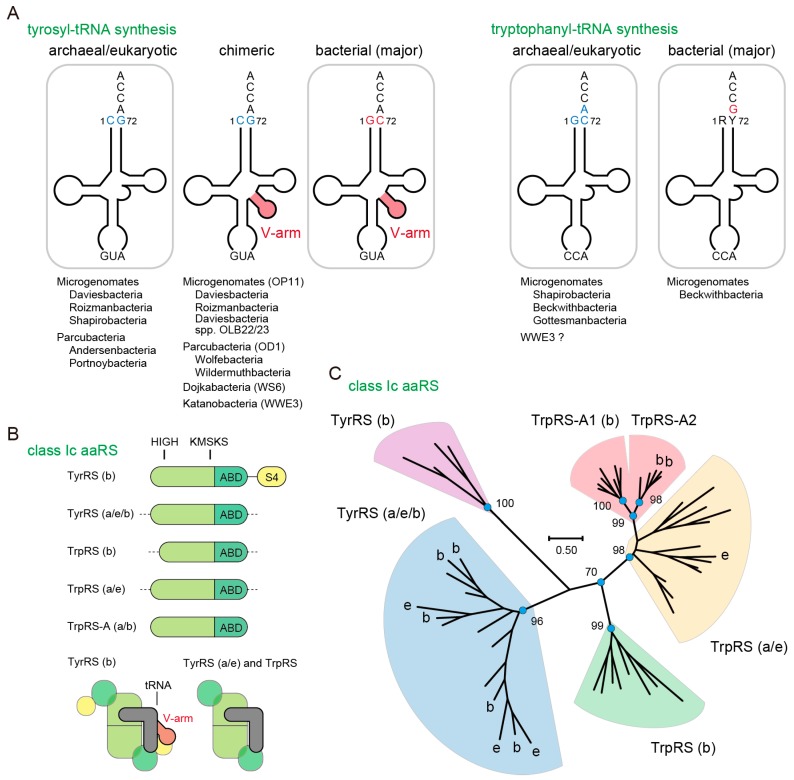
Bacteria with an archaeal tRNA^Tyr^ or tRNA^Trp^ identity. (**A**) The major identity elements for tyrosyl-tRNA synthetase (TyrRS) and tryptophanyl-tRNA synthetase (TrpRS) are shown in blue and red. Diverse subgroups of the Candidate Phyla Radiation (CPR) have archaea-like tRNA^Tyr^ and tRNA^Trp^ genes, as shown below the tRNA structures. Ca. Beckwithbacteria bacterium RBG_13_42_9 has both bacterial and archaeal pairs of TrpRS•tRNA^Trp^; (**B**) Domain structures of the class Ic aminoacyl-tRNA synthetase (aaRS) family (homodimer). The S4-like domain binds to the V-arm of bacterial tRNA^Tyr^. ABD denotes anticodon-binding domain. TrpRS-A is a newly identified TrpRS homolog. Class Ic aaRS is known to form a homodimer (in a few cases pseudo-homodimer) and binds to one tRNA molecule at a time (half-of-the-sites). Bacterial, archaeal and eukaryotic origins are indicated with b, a and e, respectively; (**C**) Phylogenetic analysis of the class Ic aaRS family. Maximum likelihood bootstrap values (100 replicates) are shown. The TrpRS-A species are split into two clades. The TrpRS-A2 proteins may chelate a [4Fe–4S] cluster.

Bacteria, archaea and eukarya share the standard genetic code, which suggests that they share a universal common ancestor (LUCA). However, the molecular systems underlying the standard genetic code are not completely conserved between all domains of life. In aminoacyl-tRNA synthesis, several elements of tRNA such as the anticodon sequence, other nucleotide residues, post-transcriptional modifications, and local and global tertiary structures are recognized by the cognate aminoacyl-tRNA synthetase (aaRS) [1,2,3]. While it is known that in all domains of life the anticodon sequences of tRNA^Tyr^ and tRNA^Trp^ are recognized by their cognate aaRSs, the other major identity elements of tRNA^Tyr^ and tRNA^Trp^ are distinct between the bacterial domain and the archaeal and eukaryotic domains [1,2,4,5] (Figure 1A). In bacteria, tRNA^Tyr^ contains a G1-C72 base pair and a variable arm (V-arm) that is recognized by the additional *C*-terminal S4-like domain of bacteria-type TyrRS (Figure 1A,B) [6]. On the other hand, archaea and eukaryotes encode a tRNA^Tyr^ lacking the V-arm and containing a C1-G72 base pair (Figure 1A) [6]. For tRNA^Trp^, bacteria encode a tRNA^Trp^ with G73, whereas archaea and eukaryotes have a tRNA^Trp^ with A73 and a G1-C72 base pair (Figure 1A) [7]. Thus, unlike the other aaRS species, archaeal and eukaryotic TyrRS and TrpRS have not been found in the bacterial domain [6,8,9,10,11,12,13,14,15].

A fundamental question in understanding the evolution of the genetic code is whether tRNA identities were established at the time of LUCA, and if so, which tRNA identity set was used [13,16]. The discovery of bacteria with an archaeal tRNA identity would provide support for the hypothesis that archaeal tRNA identity sets may have been used in LUCA. A clue to the answer to this question was provided by two synthetic biology studies [17,18]. The artificial gene transfer of an archaeal or eukaryotic TyrRS or TrpRS gene to *Escherichia coli* was successfully achieved by the simultaneous transfer of an archaeal or eukaryotic tRNA gene [17,18]. These heterologous aaRS•tRNA pairs functionally replaced the endogenous aaRS•tRNA pairs in *E. coli*. Thus, it can be hypothesized that the archaeal tRNA^Tyr^ and tRNA^Trp^ identities might have been used in LUCA.

Inspired by these studies, we carefully re-investigated the phylogenetic distribution of TyrRS and TrpRS. In the present study, archaea-, eukarya-, and bacteria-type is used to indicate the canonical archaea-, eukarya-, or bacteria-type aaRS, respectively, independent of the organism in which the enzyme is identified. Surprisingly, we found a putative bacterial species annotated to have a eukarya-type TyrRS gene (EKE14628.1) [19]. This Ca. Roizmanbacteria bacterium belongs to the candidate phyla radiation (CPR) composed of diverse uncultured bacteria which are often symbiotic with DPANN archaea [20,21,22,23,24]. Although composite genomes of CPR bacteria and DPANN archaea are sometimes contaminated by DPANN archaeal genomes and CPR bacterial genomes, respectively, a recent study was able to identify archaea-like form II/III RubisCO genes in CPR bacteria [25]. These findings prompted us to search for archaea/eukarya-type Tyr- and Trp-encoding systems in bacteria.

## 2. Materials and Methods

### Bioinformatics

Archaeal and eukaryotic TyrRS genes and non-canonical bacteria-type TyrRS genes were collected in three steps. First, TyrRS genes of representative archaea and eukaryotes were collected by a keyword search (tyrosyl/tryptophanyl-tRNA synthetase; tyrosine/tryptophan--tRNA ligase) and a BLASTp search in the National Center for Biotechnology Information (NCBI) database. Next, archaea/eukarya-type TyrRS genes in the bacterial domain were collected by a BLASTp search in the NCBI database and manually curated. Lastly, TyrRS amino acid sequences which showed about ≥40% similarity with a query sequence (GenBank: KKM02188.1) were collected from all genome, metagenome and metatranscriptome protein sequence datasets in the Integrated Microbial Genomes (IMG) system [26] (last update September 2016). The reason for employing KKM02188.1 was to find bacteria whose TyrRSs resemble opisthokontal (fungal and animal) TyrRSs. The query protein belongs to an unknown fungus in a marine sediment metagenome and shows good similarity (41%–51%) to both opisthokontal and Daviesbacteria GW2011_GWA1_38_7 TyrRS species.

Non-canonical TrpRS genes in the bacterial domain were first identified by a BLASTp search in the NCBI database using the *Pyrococcus horikoshii* TrpRS (UniProtKB: O59584.2) as query. Non-canonical TrpRS genes which showed >40% amino acid similarity with Ca. Beckwithbacteria bacterium RBG_13_42_9 TrpRS-A were collected by BLASTp searches in the NCBI database and IMG’s groundwater metagenome datasets. The obtained protein sequences were analyzed by Clustal X 2.1 [27] (for rough alignment), SeaView ver 4.0 [28] (for manual curation), MEGA7 [29] with the default settings (Maximum Likelihood, JTT model, Uniform rates, Use all Gaps/Missing sites, for phylogeny estimation), and BoxShade Server ver. 3.21 (for visualization). Multiple sequence alignment analyses by Clustal X were followed by a manual curation based on the reported structure-based alignments of TyrRS and TrpRS [30,31,32,33,34,35]. For the phylogenetic analyses of class Ic aaRSs, *N*- and *C*-terminal protein sequences were trimmed and nonconserved insertion sequences removed; up to 13 residues upstream of the HIGH motif and to the end of the anticodon binding domain were included in the analyses. Protein two- and three-dimentional structural prediction was performed using JPred 4 [36] and SWISS-MODEL [37], respectively. tRNA^Tyr^ and tRNA^Trp^ sequences were identified by a BLASTn search using automatically annotated tRNA^Tyr^ and tRNA^Trp^ sequences as queries.

## 3. Results

### 3.1. Identification of Non-Canonical Class Ic aaRS Sequences

We found tRNA^Tyr^ with C1-G72 and archaea/eukarya-type TyrRS genes in diverse subgroups of the Parcubacteria (OD1), Microgenomates (OP11), Dojkabacteria (WS6) and Katanobacteria (WWE3) phyla in CPR [22,25] (Figure 1A). In many cases, the CPR tRNA^Tyr^ species with C1-G72 contain a V-arm, indicating that these non-canonical tRNA^Tyr^ species are derived from bacterial tRNA^Tyr^ with a V-arm. Both a V-arm-containing and a V-arm-lacking tRNA^Tyr^ with C1-G72 are found in Ca. Roizmanbacteria bacterium GW2011_GWC2_34_23. On the other hand, tRNA^Trp^ with A73 and archaea/eukarya-type TrpRS genes are found in a few Microgenomates bacteria (Figure 1A). Both a bacterial tRNA^Trp^ with G73 and a tRNA^Trp^ with A73 exist in Ca. Bechwithbacteria bacterium RBG_13_42_9. We named these non-canonical TrpRS species as TrpRS-A (Figure 1B). Interestingly, TrpRS-A is slightly different from the canonical archaeal TrpRS species, but highly similar to minor DPANN archaeal TrpRS species (also named TrpRS-A) found only in the groundwater metagenomes (Figure 1C). We identified only 12 instances of TrpRS-A genes in total, suggesting an infrequent distribution of these genes in nature. These bacterial and archaeal TrpRS-A species form a terminal clade within the archaeal/eukaryotic TrpRS clade and can be grouped into two sub-clades (TrpRS-A1 and TrpRS-A2) (Figure 1C). The TrpRS-A1 proteins appear to be restricted within CPR bacteria. The TrpRS-A2 proteins are predicted to chelate a [4Fe–4S] cluster through their four cysteine residues, like some bacterial and archaeal TrpRS proteins having a C-x22-C-x6-C-x2-C motif [38].

### 3.2. Archaea/Eukarya-Type TyrRS in the Bacterial Domain

**Figure 2:**
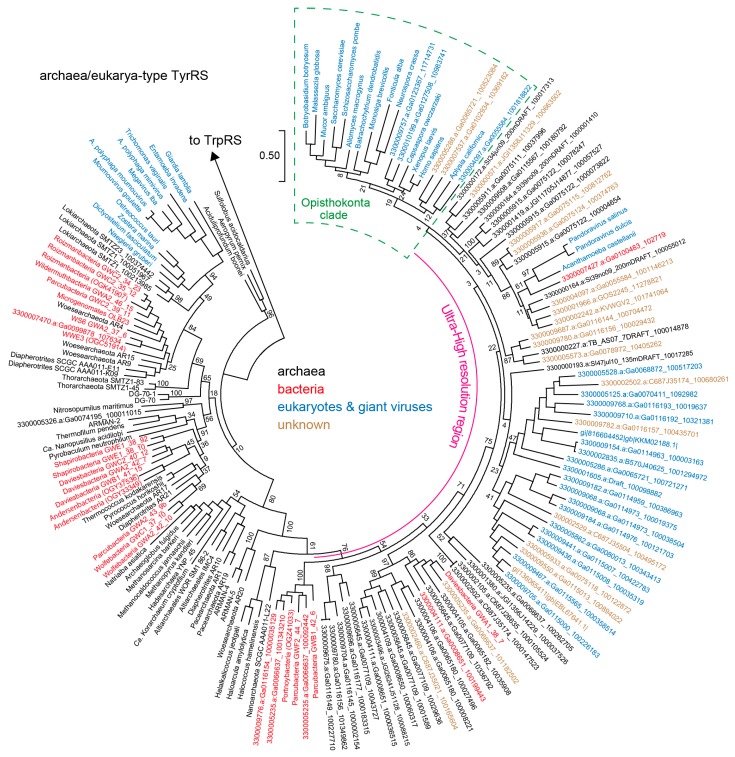
A phylogenetic tree for archaea/eukarya-type TyrRS. Bootstrap values (%) are shown for the rooted Maximum Likelihood tree made with 100 replicates using MEGA7. The ‘Ultra-High resolution’ region shows almost all TyrRS sequences identified by the comprehensive genome/metagenome/metatranscriptome analysis using gi|816604452|gb|KKM02188.1| as query for BLASTp. The archaeal species in the Ultra-High resolution region may belong to the DPANN superphylum according to the Joint Genome Institute’s annotation pipeline and our manual annotation. The opisthokontal (fungal and animal) TyrRS clade is marked with a green box. We chose a few representative TyrRS sequences for each major bacterial group (Roizmanbacteria, Daviesbacteria, Shapirobacteria, Wolfebacteria and Andersenbacteria) after confirming the sequence similarity within the same group. In contrast, we identified three orphan TyrRS genes belonging to bacteria in the Ultra-High resolution region. TrpRS sequences of *Thermus thermophilus* (bacteria) and *Pyrococcus horikoshii* (archaea) were used as an outgroup.

**Figure 3:**
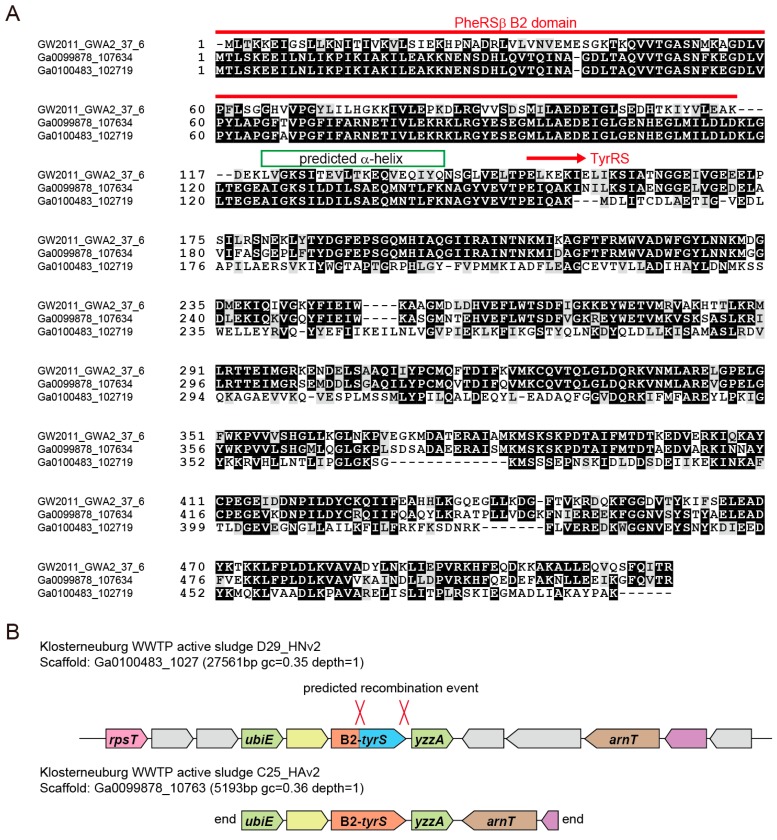
The TyrRS species with a *N*-terminal B2 domain fusion. (**A**) Multiple sequence alignment of the three B2-TyrRS proteins; (**B**) The genomic loci of the two B2-TyrRS genes in active sludge metagenomes. Predicted recombination sites are indicated.

We then investigated the collected TyrRS sequences. A high-resolution phylogeny for archaea/eukarya-type TyrRS suggests that several lineages of CPR bacteria independently obtained an archaea/eukarya-type TyrRS gene from archaea (Figure 2). Alternatively, lateral gene transfer (LGT) might have occurred from bacteria to archaea and other groups of bacteria. Interestingly, one bacterial TyrRS sequence (3300007427.a:Ga0100483_102719) is highly similar to the TyrRS sequences of *Acanthamoeba castellanii* and Pandoraviruses (the Eukarya domain) (see the Ultra-High resolution region in Figure 2). Thus, LGT between bacteria and amoeba or giant viruses can be reasonably assumed. This Pandoravirus-like TyrRS, as well as two other TyrRSs derived from WS6 bacterium GW2011 GWA2_37_6 [24,39] and an active sludge metagenome, possess a B2 domain of bacterial phenylalanyl-tRNA synthetase β-subunit (PheRSβ) [40]. This domain is fused to the *N*-terminus of the TyrRS by a long α-helix (Figure 3A). The B2 domain belongs to the RNA-binding OB folds, but it is missing in many CPR-bacterial PheRSβ (for example, OGE14653.1). We also found that a few Microgenomates lysyl-tRNA synthetases (KKR67068.1 & KKQ91124.1) have an additional *C*-terminal domain that is very similar to this B2 domain and predicted α-helix. It is known that aaRS proteins are often fused with an OB domain [41]. The two Klosterneuburg active sludge metagenomic contigs containing the B2-TyrRS fusion genes showed almost identical gene organization and gene sequences, indicating that these two contigs belong to two closely-related bacteria. However, only the TyrRS ‘domain’ sequences are different in terms of sequence similarity (Figure 3A,B). While one is Pandoravirus-like, as previously mentioned, the other is most similar to the TyrRS ‘domain’ sequence of the B2-TyrRS species of WS6 bacterium GW2011 GWA2_37_6 (Figure 2 and Figure 3A). A possible explanation for this is that the TyrRS ‘domain’ sequence was replaced with the Pandoravirus-like sequence in a bacterial lineage (Figure 3B, the upper contig). This finding will help us understand aaRS evolution through domain fusion and swapping.

### 3.3. Non-Canonical TrpRS Species and Their Associating Proteins

**Figure 4:**
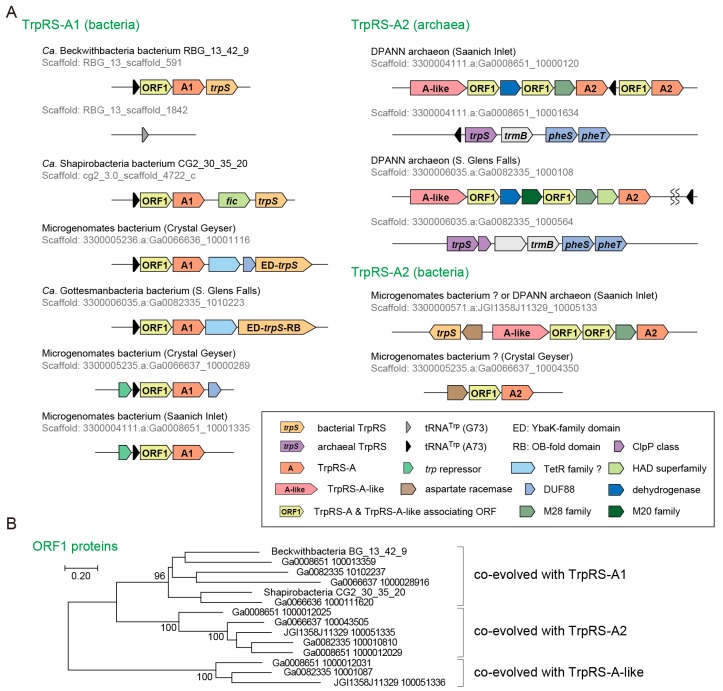
Non-canonical TrpRS species. (**A**) The genetic loci and the operon structures of TrpRS-A genes. The origins of these uncultured organisms are described in the parentheses and indicated with “CG” (Crystal Geyser groundwater) and “RBG” (Rifle BackGround groundwater). In a few Microgenomates species, TrpRS is fused with small proteins; (**B**) Co-evolution of the ORF1 genes with the TrpRS-A and TrpRS-A-like genes. Bootstrap values (%) are shown for the unrooted Maximum Likelihood tree made with 100 replicates using MEGA7.

We then investigated the genetic loci of the TrpRS-A genes (Figure 4A). In bacteria, tRNA^Trp^ (A73) and TrpRS-A comprise an operon. Interestingly, this operon is either followed by a bacteria-type TrpRS gene or headed by a *trp* repressor gene (Figure 4A). This is consistent with Ca. Beckwithbacteria bacterium RBG_13_42_9 possessing a bacterial tRNA^Trp^ (G73) somewhere else in the genome. The observed operon structures indicate that the tRNA^Trp^ (A73) and TrpRS-A1 genes may be regulated by tryptophan availability and could coexist with a canonical bacterial TrpRS•tRNA^Trp^ pair. In archaea, one or two tRNA^Trp^ (A73) genes, one or two TrpRS-A2 genes and another gene encoding a TrpRS-A homolog (named TrpRS-A-like) are found in addition to a single canonical archaeal TrpRS gene. TrpRS-A-like has several insertions and deletions compared to archaea/eukarya-type TrpRS and TrpRS-A. Thus, TrpRS-A and TrpRS-A-like coexist with a canonical archaeal TrpRS in archaea. Interestingly, TrpRS-A2 and TrpRS-A-like genes are also found in putative bacterial metagenomic contigs (Figure 4A), implying multiple LGT events.

**Figure 5:**
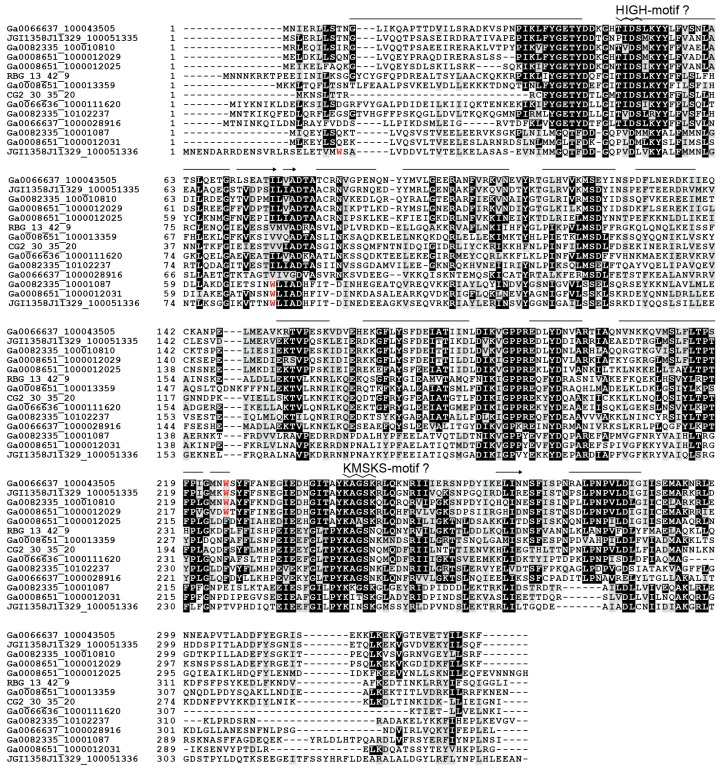
Multiple pairwise alignment of the ORF1 proteins and their structural prediction by SWISS-MODEL. Bars and arrows above the amino acid sequences represent predicted α-helices and β-strands, respectively, whereas zigzags indicate predicted HIGH and KMSKS motifs. The predicted overall structure suggests that the ORF1 protein might be a homolog of class I aaRS. Tryptophan (W) residues are shown as red letters.

We found an unusual open reading frame (named as ORF1) between the tRNA^Trp^ (A73) and TrpRS-A1 genes in the bacterial operons and in the gene clusters of the TrpRS-A2 and TrpRS-A-like in archaea (Figure 4A). The ORF1 is completely conserved and appears to have co-evolved with the TrpRS-A1, TrpRS-A2 and TrpRS-A-like ORFs (Figure 4B). ORF1 sequences show no significant similarity with any known protein (less than score 40 in NCBI BLASTp searches). However, the predicted structure of the ORF1 proteins includes HIGH-motif-like and KMSKS-motif-like motifs [2], as well as many α-helices, thereby suggesting that these proteins might be structural homologs of class I aaRSs (Figure 5). Seventy residues from the putative HIGH motif of ORF1 matched the corresponding region of cysteinyl-tRNA synthetase (a class Ia aaRS) with 16.9% sequence identity in our SWISS-MODEL prediction. However, in the ORF1 structure prediction, the Rossmann-fold domain is missing [42], indicating a function distinct from class I aaRS or class I aaRS homolog that synthesizes cyclo(l-Trp-l-Trp) using tryptophanyl-tRNA^Trp^ [43]. Interestingly, both TrpRS-A1 and ORF1 pairs and bacteria-type TrpRSs of these bacteria lack any tryptophan residues (see Figure 5 for the ORF1 cases). This might imply that tryptophan may be limited for these bacteria living in groundwater environments.

## 4. Discussion

It was thought that LGT of some archaeal/eukaryotic aaRS genes to bacteria would be prevented by the difference in tRNA identity rules. A previous bioinformatic study detected a case of eukaryotic-like histidine (His) tRNA identity (i.e., A73) in certain α-proteobacteria [12]. These α-proteobacterial tRNA^His^ species have A73 and lack G-1 [44]. However, a subsequent biochemical study revealed A73 to be a minor identity element of the *Caulobacter crescentus* histidyl-tRNA synthetase (HisRS) [45]. A recent comprehensive bioinformatic study did not support any LGT event of HisRS from eukaryotes to α-proteobacteria [8], suggesting that the eukaryotic-like tRNA^His^ identity in certain α-proteobacteria might be a result of convergent evolution. Similarly, it is known that some mitochondrial aaRSs violate the bacterial identity rules. For example, human mitochondrial TyrRS charges mitochondrial tRNA^Tyr^ species with G1-C72 (wild-type) or C1-G72 (mutant) with the same efficiency [46]. The V-arm is missing in the human mitochondrial tRNA^Tyr^, whereas the human mitochondrial TyrRS retains the S4-like domain [47].

In contrast to these previous findings, our results provide the first evidence that archaeal or eukaryotic TyrRS and TrpRS genes exist in a few lineages of bacteria that have tRNA^Tyr^ or tRNA^Trp^ species with archaeal and eukaryotic identity elements. Intimate relationships between CPR bacteria and DPANN archaea may have facilitated LGT of TyrRS and TrpRS genes. Furthermore, our high-resolution TyrRS phylogeny and another comprehensive study [8] revealed that the LGT of TyrRS may have occurred several times among DPANN archaea, CPR bacteria, eukaryotes and giant viruses (Figure 2). Taken together, our data make clear that tRNA identities may not be hardwired to each domain of life.

The functions of TrpRS-A, TrpRS-A-like and the ORF1 proteins remain unclear. Their operon structures suggest that they are involved in tryptophan metabolism and encoding, rather than producing complex antibiotics, plant toxins and peptidoglycans [43,48]. Since TrpRS-A and TrpRS-A-like genes appear to occur in addition to the canonical TrpRS gene in both bacteria and archaea, TrpRS-A and TrpRS-A-like genes might encode additional copies of TrpRS that display a higher or lower specificity for tryptophan than the canonical TrpRS in order to confer antibiotic resistance [49] or to cope with stress [50]. The ORF1 proteins might bind to the homodimers of TrpRS-A and TrpRS-A-like in order to be stabilized in a complex [51]. Another possibility is that TrpRS-A and TrpRS-A-like proteins might form heterodimers with the partner ORF1 protein. It is known that some eukaryotes have a double-length TyrRS species forming a pseudo-dimer in which one of the “subunits” is catalytic but has lost the affinity for the tRNA^Tyr^ anticodon, whereas the other is non-catalytic but still recognizes the anticodon [52]. In addition, trans-oligomerization of duplicated threonyl-tRNA synthetases is known [50]. Future studies should elucidate the biochemical properties and the biological functions of these proteins.

## Abbreviations

The following abbreviations are used in this manuscript:

CPR	candidate phyla radiation
aaRS	aminoacyl-tRNA synthetase
TyrRS	tyrosyl-tRNA synthetase
TrpRS	tryptophanyl-tRNA synthetase
PheRS	phenylalanyl-tRNA synthetase
HisRS	histidyl-tRNA synthetase
ORF	open reading frame
LGT	lateral gene transfer
